# Cardiac manifestations in children and adolescents diagnosed with pediatric multisystem inflammatory syndrome related to COVID-19

**DOI:** 10.1016/j.jped.2025.101461

**Published:** 2025-10-31

**Authors:** Thiago T. Panizzi, Katharine A. de Souza, Gabriela B. Stutz, Fernanda M.C.F. Lemos, Marta C.F. Rodrigues, Rozana G. de Almeida, Luciane A. da Rocha, Flavio R. Sztajnbok, Adriana R. Fonseca, Nathalie J.M. Bravo-Valenzuela

**Affiliations:** a Universidade Federal do Rio de Janeiro, Instituto de Puericultura e Pediatria Martagão Gesteira, Rio de Janeiro, RJ, Brazil; b Universidade Federal do Amazonas, Programa de Pós Graduação em Ciências da Saúde, Manaus, AM, Brazil

**Keywords:** COVID-19, MIS-C: multisystem inflammatory syndrome in children, Cardiovascular abnormalities, Coronary circulation, Echocardiography, Global longitudinal strain

## Abstract

**Objectives:**

To describe clinical and cardiologic findings in patients with multisystem inflammatory syndrome in children (MIS-C) in a follow-up of up to 3 years.

**Materials and methods:**

A retrospective-prospective, observational, longitudinal study was conducted, including children and adolescents up to 18 years diagnosed with MIS-C (WHO criteria), at a university center between March 2020 and December 2024. Demographic, clinical, and laboratory data, electrocardiograms, and transthoracic echocardiograms were analyzed at admission and at 12 months and left ventricular global longitudinal strain (LV-GLS) three years after diagnosis. Statistical analysis used frequencies for categorical variables, and means with standard deviations or medians with interquartile ranges for continuous variables. Differences in proportions between patients with and without cardiovascular abnormalities were assessed using Fisher's exact test, Chi-squared, or Wilcoxon rank-sum test (significant p-value < 0.05). T-test was used to compare left ventricular ejection fraction (LVEF) and coronary artery Z scores.

**Results:**

Thirty-six patients were included (males 69.4%), with a median age at diagnosis of 2.15 years (IQR 3.3). At admission, 41.7% presented with clinical or echocardiographic abnormalities, which were absent at 1-year follow-up. However, three years after diagnosis, among the 11 patients evaluated with LV-GLS, 10 showed changes indicating subclinical dysfunction not detected by conventional electrocardiogram or echocardiography.

**Conclusion:**

Clinical and echocardiographic cardiovascular abnormalities are common in the acute phase of MIS-C. Although most patients showed clinical and echocardiographic resolution, LV-GLS proved valuable for detecting subclinical myocardial dysfunction not identified by conventional evaluation, highlighting its potential as a screening tool in short- and long-term follow-up.

## Introduction

In April 2020, the first cases of a new clinical presentation of COVID-19 in children showing similarities with Kawasaki disease (KD), a vasculitis that mainly affects medium-caliber arteries, such as the coronary arteries, were reported. The condition, known as multisystem inflammatory syndrome in children (MIS-C), emerged 2–4 weeks after local peaks of acute COVID-19 cases [1,2]. Children with MIS-C often present severe gastrointestinal and neurological symptoms, higher frequency of shock, arrhythmias, and ventricular dysfunction, lower platelet and lymphocytes counts, and higher C-reactive protein (CRP) levels than classic KD [3]. They can also develop coronary aneurysms without the classical signs of KD. MIS-C is widely recognized for its potential for cardiovascular involvement, ranging from subclinical abnormalities — including elevated troponin, asymptomatic arrhythmia, and imaging changes — to overt manifestations including heart failure, myocarditis, pericarditis, coronary vasculitis, and valvulitis [4, 5, 6].

Early studies suggested that these manifestations are predominantly transient, with complete recovery after treatment [7, 8, 9, 10]. However, variability remains regarding the frequency and severity of cardiovascular changes, with some studies [11] reporting higher rates of coronary dilation and ventricular dysfunction, and most follow-up studies are limited to one year, leaving the long-term trajectory of cardiac involvement poorly understood. Moreover, subclinical markers of myocardial dysfunction, such as LV-GLS, may reveal abnormalities not detectable by conventional echocardiography [12,13].

Importantly, no previous studies have combined long-term follow-up beyond one year with LV-GLS assessment. By extending follow-up to three years and incorporating LV-GLS measurements, the present study aims to fill this gap and provide new insights into the persistence of subclinical myocardial dysfunction in MIS-C.

Considering the importance of cardiovascular disease as a cause of morbidity and mortality in MIS-C, identifying predictors of cardiac dysfunction is extremely relevant. The objective of this study was to follow clinical and cardiologic parameters in children and adolescents with MIS-C through transthoracic echocardiography and clinical assessment, at admission, 12 months, and three years after the diagnosis, including LV-GLS measurements.

## Materials and methods

### Study design

**Figure 1:**
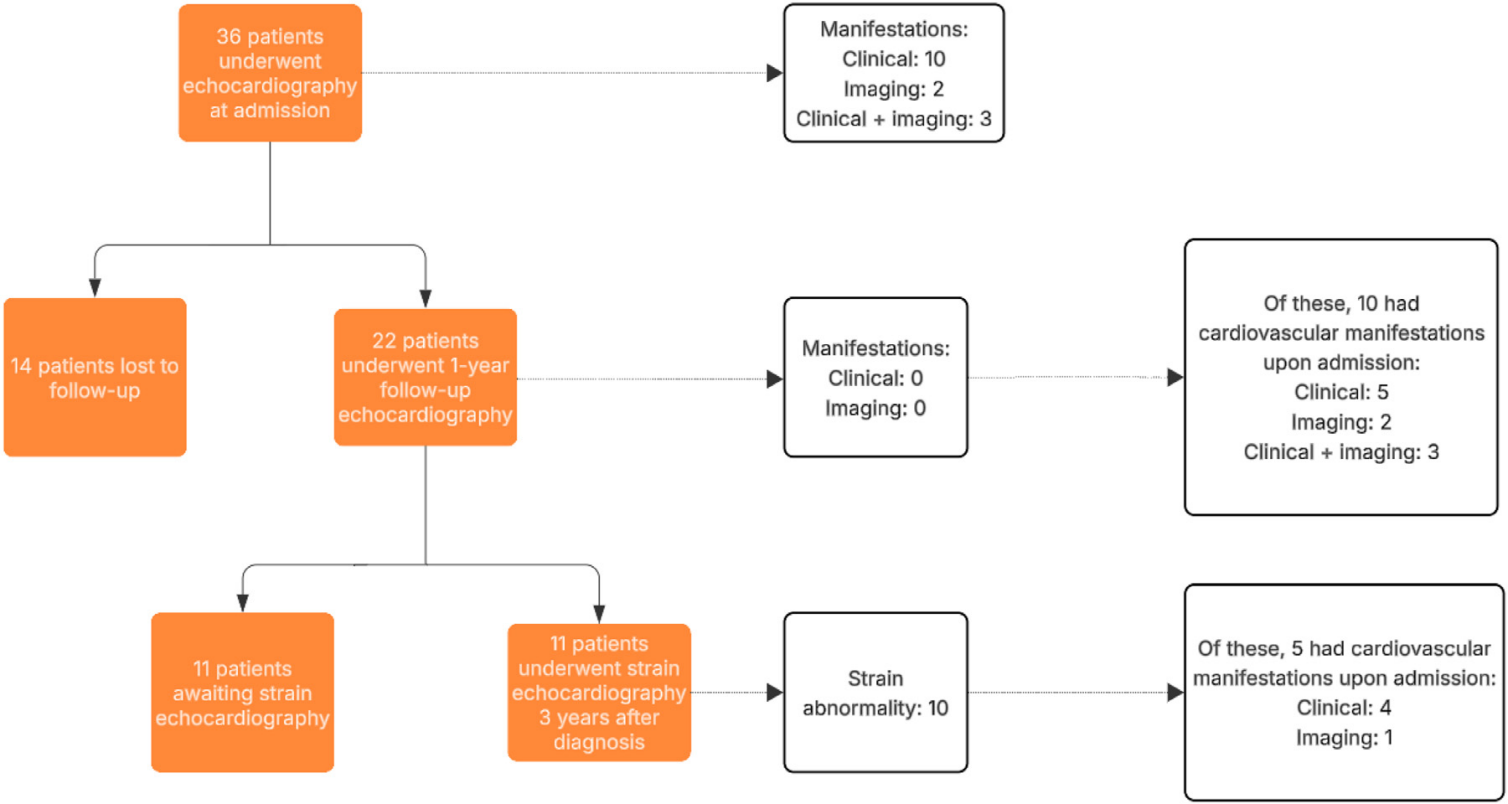
Flow diagram of the study population.

This was a retrospective and prospective, observational, longitudinal, and analytical study, including children and adolescents aged 0 to 18 years diagnosed with MIS-C at a specialized university center. In the retrospective phase (March 2020-July 2022), data were collected through medical records. In the prospective phase, July 2022-December 2024, patients were followed up with clinical evaluation and transthoracic echocardiography. Between December 2024 and June 2025, patients were evaluated by LV-GLS. Figure 1 shows the flow diagram of patients evaluated at admission, 1 year, and 3 years after symptom onset.

### Ethical considerations

The study was approved by the institution’s Research Ethics Committee.

### Inclusion and exclusion criteria

The sample was collected by convenience sampling from patients consecutively diagnosed with MIS-C, according to WHO criteria [14], during the study period. Patients with a previous diagnosis of congenital heart disease were excluded.

Data were recorded in a standardized instrument: sex, birth date, disease onset, hospitalization/discharge dates, date of diagnosis, comorbidities, microbiological data, vaccination status, vital signs, clinical and laboratory findings, electrocardiographic and echocardiographic findings, treatment, complications, and outcomes.

### Vital signs/constitutional symptoms at admission and follow-up

hypotension/hypertension (adjusted for age, sex, and height), tachycardia, bradycardia, oxygen saturation < 95 %, peripheral hypoperfusion/shock, prostration, irritability, and weight loss.

### Clinical manifestations on admission

Fever, duration of fever (days), clinical findings (mucocutaneous, musculoskeletal, gastrointestinal, lymphoid organ, cardiovascular, respiratory, neurological, and genitourinary). Arrhythmias, hypertension, hypotension/shock, heart failure, pericarditis, and thrombosis were considered cardiovascular clinical changes.

### Laboratory tests at admission

Routine laboratory tests included complete blood count, C-reactive protein (CRP), erythrocyte sedimentation rate (ESR), D-dimer, international normalized ratio (INR), activated partial thromboplastin time (aPTT), fibrinogen, ferritin, lactate dehydrogenase (LDH), urea and creatinine levels, albumin, AST (aspartate aminotransferase), ALT (alanine aminotransferase), and electrolytes. Values were classified as normal/abnormal according to the laboratory reference.

### Cardiovascular examinations

Electrocardiograms (ECGs) were performed by a qualified technician and evaluated by a pediatric cardiologist and classified as normal or abnormal, with abnormalities including: tachyarrhythmia, prolonged PR interval, prolonged QT interval, bundle branch block, atrioventricular block, ST-segment alteration, left atrial enlargement, right atrial enlargement, left ventricular and right ventricular enlargement.

Echocardiograms were performed in the Pediatric Cardiology Department at the center using a Toshiba Aplio 300 device with 3–7.5 MHz probes, operated by a specialized pediatric cardiologist, at admission and 12 months after diagnosis. Parameters included LVEF, pericardium, cardiac valves, and left/right coronary artery diameters. LVEF was considered abnormal when ≤ 55 %, and coronary arteries were considered ectatic when Z scores were > +2.

Additionally, the following echocardiographic parameters were measured at 1-year follow-up: tricuspid annular plane systolic excursion (TAPSE), mitral annular plane systolic excursion (MAPSE), and E/E’ ratio for the definition of cardiac dysfunction, considered abnormal when the Z score of E/E’ ratio > + 2 or the Z score of TAPSE or MAPSE < −2. LV-GLS by speckle tracking was assessed in a sample of 11 patients, 3 years after diagnosis. The normal reference range adopted for the LV-GLS was −18 % to −22 %, with values > −18 % considered abnormal (indicating subclinical myocardial dysfunction).

### Statistical analysis using SPSS software (IBM, 2015)

Analysis was descriptive, with absolute and percentage frequencies for categorical variables, as well as means and standard deviations for variables with normal distribution, or medians and interquartile ranges for variables with non-normal distribution. Patients with and without cardiovascular alterations were compared. Cardiovascular alterations were defined as the presence of arrhythmias, hypertension, hypotension/shock, heart failure, pericarditis/pericardial effusion, valvulitis, thrombosis, and coronary artery enlargement. Differences between proportions of those two groups were assessed using Fisher's exact test, Chi-squared test, or Wilcoxon rank-sum test. Statistical significance was defined as *p* < 0.05. The T test was used to compare LVEF and coronary Z scores. To assess the standardization and accuracy of the measurements, the intraclass correlation coefficient (ICC) was calculated using the Two-Way Random Effects model to evaluate intraobserver and interobserver agreement for coronary artery diameter measurements (20 % of the total sample).

## Results

A total of 36 patients were included (69.4 % males), with a median age at diagnosis of 2.15 years (IQR 3.3). Cardiovascular findings were observed in 15 patients (41.7 %) during the acute phase, either clinically or on echocardiography. At the 3-year follow-up, among the 11 patients assessed by LV-GLS, 10 (90.9 %) showed subclinical myocardial dysfunction, despite being asymptomatic.

### Patients’ characteristics

Thirty-six patients were included, with a male predominance (69.4 %). The median age at diagnosis was 2.15 years (range 0.5 - 13, IQR 3.3). All cases had fever, with a median total duration of 10 days (IQR 7). Comorbidities were identified in seven patients (19.4 %), mainly asthma and being overweight. All patients were hospitalized, with a median length of stay of 5 days (IQR 5), and 4 of them (11.1 %) required admission to the ICU (mean length of stay 7.5 ± 3.51 days). Patients’ characteristics are displayed in Table 1.

**Table 1:** Characteristics of the MIS-C patients included.

Characteristics	Total patients (*n* = 36)
Sex: n, ( %)	
Male	25 (69.4)
Female	11 (30.6)
Age at diagnosis years: median (IQR)	2,15 (3.3)
Comorbidities: n, ( %)	7 (19.4)
Asthma	2 (5.6)
Overweight	2 (5.6)
ASD	1 (2.8)
Pelvicalyceal dilatation	1 (2.8)
Previous vaccination for SARSCOV2	0 (0)
Hospitalization: n ( %)	36 (100)
Length of hospital stay days: median (IQR)	5 (5)
ICU admission: n ( %)	4 (11.1)
ICU stay days: average (±SD)	7.5 ± 3.51
Total duration of fever days: median (IQR)	10 (7)
COVID status: n, ( %)	
Suspected contact	25 (69.4)
IgG+	14/18 (77.8)
IgM +	2/12 (16.6)
RT-PCR +	3/25 (12)
Antigen test +	2/27 (7.4)

Values are presented as n ( %) for categorical variables. To assess the distribution of continuous variables, the Shapiro-Wilk normality test was applied. Median with an interquartile range was used for variables with non-normal distribution and mean ± SD for variables with normal distribution.

n - number of individuals; SD – Standard Deviation; IQR – Interquartile range; ASD – autism spectrum disorder; ICU – Intensive Care Unit; RT-PCR – Reverse Transcription Polymerase Chain Reaction; IgM – Immunoglobulin M; IgG – Immunoglobulin G.

### Clinical characteristics

During the acute phase, eight cases presented tachycardia (22.2 %), eight prostration (22.2 %), and five hypotension (13.9 %). Fever was the most prevalent clinical manifestation (100 %), followed by mucocutaneous (88.9 %) and respiratory manifestations (75 %). Clinical cardiovascular manifestations accounted for 36.1 % of the cases. Table S1 describes the main clinical findings in the included patients.

### Laboratory data

D-dimer was increased in 90.3 % of the tested cases, followed by an increase in LDH (82.1 %), CRP (77.8 %), ESR (75.8 %), and ferritin (65 %) values. Other laboratory findings are described in Tables S2 and S3.

### Cardiovascular manifestations

Nine patients had ECG abnormalities. Sinus tachycardia (after excluding other causes such as fever) was the most prevalent arrhythmia (22.2 %), followed by first-degree atrioventricular block (2.8 %) and left atrial enlargement. (2.8 %). In total, 15 patients (41.7 %) presented cardiovascular alterations either clinically or on echocardiography. At admission echocardiography, 3 patients (8.3 %) had pericardial effusion, 2 (6.1 %) had LVEF ≤ 55 %, and 1 (3.7 %) had left coronary artery dilation. At the one-year follow-up visit, none of the cases had LVEF ≤ 55 % or coronary artery dilation. However, there was an increase in TAPSE in 7 cases (33.3 %) and MAPSE in 11 cases (52 %) compared to the age-referenced values. There was no reduction in MAPSE, TAPSE or increase in the E/E’ ratio during this period in relation to the expected Z score for the general population. Only one case presented sequelae at the end of follow-up — testicular infarction — without any other cardiovascular abnormalities. Clinical, electrocardiographic, and cardiovascular imaging findings at admission are shown in Tables 2, S4 and S5.

**Table 2:** Echocardiographic findings at admission and at one-year follow-up.

Echocardiographic (echo) findings	Admissional echo	At 1-year follow-up
LVEF ≤ 55 %: n, ( %)	2/33 (6.1)	0/22 (0)
Pericardial effusion: n, ( %)	3/36 (8.3)	0/22 (0)
Left coronary artery Z score: mean (±SD)	−0.52 ± 1.2	−0.91 ± 1.1
Reduced: n, ( %)	1/27 (3.7)	4/20 (20)
Enlarged: n, ( %)	1/27 (3.7)	0/20 (0)
Right Coronary Artery Z score: mean (±SD)	−0.76 ± 0.87	−1.2 ± 0.91
Reduced: n, ( %)	2/27 (7.4)	6/20 (30)
Enlarged: n, ( %)	0/27 (0)	0/20 (0)

Values are presented as n ( %) for categorical variables.

To assess the distribution of continuous variables, the Shapiro-Wilk normality test was applied. Median with an interquartile range was used for variables with non-normal distribution and mean ± SD for variables with normal distribution.

n - number of individuals; SD – Standard Deviation; LVEF – Left Ventricular Ejection fraction.

### Treatment

All patients received acetylsalicylic acid (ASA), with a median onset of 10.5 days (IQR 10). Of these patients, 29 patients (80.6 %) received intravenous human immunoglobulin (IVIG), with a median of onset of 8 days (IQR 7), 25 patients (69.4 %) received oral, and 15 (41.7 %) intravenous corticosteroids. Treatment data are exhibited in Table S6.

### Comparison of echocardiogram findings at admission and after one year of follow-up

There was no statistically significant difference between the two time points, with p-values of 0.3 for LVEF, 0.6 for the left coronary artery, and 0.8 for the right coronary artery. In the intra- and inter-observer analysis, no statistically significant differences were found between the measurements, with a high degree of agreement: 0.87 for inter-observer and 0.93 for intra-observer mean values. The mean differences and the respective limits of agreement were evaluated using the Bland-Altman plot, as shown in Figure S1.

### Comparison between groups with and without cardiovascular involvement

Spearman's test did not demonstrate any statistically significant difference between the time to initiation of the main therapies used and the presence of cardiovascular abnormalities. Results are shown in Tables S7 and S8.

### Assessment of Left Ventricular Global Longitudinal Strain after 3 Years of diagnosis

Among the 11 patients recruited for LV-GLS analysis, abnormalities were observed in 10 cases (90.9 %). All patients evaluated with LV-GLS were asymptomatic at the time of assessment. The evaluation was performed on average 3.6 ± 0.6 years after diagnosis. The only case with preserved LV-GLS was a child whose mother received the COVID-19 vaccine during pregnancy. Only one patient had an abnormal initial echocardiogram characterized by coronary artery dilation. Two patients had sinus tachycardia on admission electrocardiogram; one of them developed hypotension and hypoperfusion requiring ICU admission, ventilatory support, and inotropic therapy. It is noteworthy that the patient diagnosed with testicular infarction was not included in the strain evaluation.

## Discussion

In this study, the authors described the clinical characteristics and cardiovascular outcomes of children with MIS-C at a referral center. Approximately 40 % presented cardiovascular abnormalities at admission, either clinically or echocardiographically, which were absent at one-year follow-up, with no significant changes in the LVEF or coronary Z scores between admission and one year. However, 3 years after diagnosis, 10 out of 11 patients assessed by LV-GLS showed subclinical myocardial dysfunction, despite being asymptomatic and having normal conventional echocardiograms. This finding highlights LV-GLS as the central contribution of the present study, emphasizing its utility in detecting persistent subclinical myocardial injury that would otherwise remain undetected.

Male predominance was observed, consistent with the literature [15]. The median age at diagnosis in the present study was significantly lower (2.15 years versus 9.6 years), which may reflect local demographic factors, such as a younger population pyramid, resulting in a higher proportion of cases among younger age groups. Additionally, specific characteristics of referral patterns to the specialized center may have favored the inclusion of younger children with severe or atypical presentations. Furthermore, the COVID-19 vaccination schedule in Brazil may have contributed to this difference: vaccination began with adolescents in 2021 [16], extended to children 5–11 years old in 2022 [17], and incorporated into the routine childhood immunization schedule in January 2024 for children aged 6 months-5 years [18]. Fully vaccinated children have a lower risk of developing severe forms of the disease, and a similar protective role has been observed in reducing complications, particularly MIS-C [19].

Regarding comorbidities, the present findings are also similar to those of Bulut [15], with a predominance of asthma and obesity, as well as similarities in the pattern of cardiovascular and gastrointestinal involvement, although the authors observed a higher prevalence of mucocutaneous and respiratory manifestations in the present cohort. Several studies have shown that laboratory markers are associated with the severity of MIS-C [20,21]. Lymphopenia, neutrophilia, anemia, and thrombocytopenia, as well as significantly high inflammatory markers (CRP, ESR) and liver transaminases, elevations in cardiac biomarkers, such as troponin, BNP, and pro-BNP, are frequently reported [1, 2, 3, 4, 5, 6,22]. Although the present study did not include the assessment of cardiac biomarkers such as troponin, BNP, and pro-BNP, a high frequency of increased inflammatory markers was observed.

The electrocardiographic findings observed in the cohort are similar to those reported in the literature, though with a lower prevalence. A scoping review published by Broberg et al. [23] showed that approximately 70 % of MIS-C patients present electrocardiographic abnormalities, compared to 35.3 % in the study by Valverde [6] and 25 % in the present study. The lower frequency of electrocardiographic changes in the studied cohort may be related to differences in the clinical characteristics or stricter diagnostic criteria. These findings underscore the importance of systematic ECG monitoring in these patients, considering the potential for myocardial involvement and the risk of progression to ventricular dysfunction or arrhythmias.

Initially, MIS-C cardiac manifestations were considered to be transient and without long-term consequences. Minocha et al. demonstrated that, although 73 % of patients had cardiac abnormalities at the time of hospital admission, all findings returned to normal values after discharge [7]. Subsequent studies also observed transient myocardial dysfunction in patients with MIS-C with complete recovery of cardiac function when treatment was appropriately instituted [8,9]. In 2023, Kapoor et al. conducted a retrospective observational study with 54 children diagnosed with MIS-C and reported normal echocardiograms at six months and one year of follow-up [10]. The initial findings are consistent with this, as no obvious echocardiographic abnormalities were observed after one year of follow-up. Wu et al. showed that 13–26 % of MIS-C patients presented coronary artery dilation, whereas in the present cohort, this was identified in only one patient (3.7 %). The same narrative review describes ventricular dysfunction as a common finding in MIS-C, with rates ranging from 33–50 %, contrasting with 6.1 % observed in the present study [11].

The treatment and follow-up of these patients at the university referral center, the use of ASA and IVIG in the vast majority of patients, may partially explain the lower frequency of cardiovascular findings observed in this cohort (41.7 %), as well as the low incidence of long-term sequelae, limited to a single case of testicular infarction. The present findings align with the observations made by Phirtskhalava et al. and Shah et al., who indicated that most children experienced complete clinical and myocardial recovery when treated appropriately. This evidence reinforces that timely and appropriate therapeutic management is directly associated with overall positive recovery [24,25].

However, recent evidence suggests persistent subclinical myocardial injury, even with normal conventional echocardiograms. The authors identified a high prevalence of later LV-GLS abnormalities despite preserved LVEF, suggesting the presence of subclinical myocardial dysfunction. Anagnostopoulou et al. identified LV-GLS abnormalities in 42.9 % of patients at admission, but they persisted in only one of them [12]. De Wolf et al. observed decreased LV-GLS in 35 % of patients during follow-up with a mean time interval of 12.1 ± 5.8 months [26]. In the general population, LV-GLS is an independent predictor of cardiovascular morbidity and mortality, providing additional prognostic information beyond current risk stratification models, both for composite cardiovascular outcomes and specifically for heart failure. As an early marker of cardiac dysfunction, LV-GLS can identify individuals at particularly high risk of cardiovascular events [27]. However, this assessment remains limited in the pediatric population, with a scarcity of data confirming its prognostic value and clinical utility in this age group. In this cohort, LV-GLS abnormalities primarily reflect subclinical myocardial dysfunction in asymptomatic children. The persistence of this alteration reinforces the importance of prolonged cardiac monitoring after the acute phase of the disease, as reported by other authors [13,28], which also highlights the usefulness of speckle tracking echocardiography as a sensitive tool for the early detection of subclinical myocardial dysfunction.

The mechanisms underlying persistent LV-GLS abnormalities in MIS-C are not yet fully understood. Possible contributors include residual myocardial inflammation, microvascular dysfunction, and subtle myocardial fibrosis that may persist after clinical recovery. These processes could account for the subclinical myocardial dysfunction detected by strain imaging, highlighting the relevance of long-term cardiovascular surveillance in this population.

It is also important to highlight that, although reduced TAPSE and MAPSE values are classically associated with ventricular dysfunction, the authors observed increased measurements in some patients at one-year follow-up. These values exceeded reference ranges, possibly reflecting a compensatory response or hyperdynamic cardiovascular state, possibly related to prior MIS-C inflammation. Although these findings do not indicate overt myocardial dysfunction, they underscore the complexity of cardiac involvement post-MIS-C and support the importance of longitudinal follow-up with detailed functional assessment, especially through more sensitive techniques such as strain imaging.

### Limitations

A high rate of loss to follow-up was observed, attributed to the rapid resolution of clinical and laboratory manifestations in most patients, which resulted in a smaller number of patients undergoing serial cardiological evaluations. Additionally, the unavailability of troponin and pro-BNP tests at the institution during the study period prevented the inclusion of these markers in the analysis. Similarly, echocardiographic measurements of TAPSE, MAPSE, and E/E’ ratio were not widely performed during admission exams due to the lack of standardized echocardiographic protocols at the beginning of the pandemic. Finally, the LV-GLS measurement was not performed in the initial follow-up of patients because of technical issues. Only 11 patients underwent LV-GLS assessment at the three-year follow-up, representing a small sample size, which limits the generalizability of these findings. Therefore, these results should be interpreted as hypothesis-generating, highlighting the need for larger prospective studies to confirm the persistence and clinical significance of subclinical myocardial dysfunction in children with MIS-C.

## Conclusions

Clinical or echocardiographic cardiovascular abnormalities are common findings in the acute phase of MIS-C, highlighting the importance of thorough and comprehensive cardiological evaluation during the acute phase of the disease. In this study, most patients showed resolution without sequelae in subsequent echocardiographic exams. In this context, the analysis of LV-GLS by speckle tracking echocardiography has proven to be a sensitive and promising tool for identifying subclinical myocardial dysfunctions that would not be detected by parameters such as LVEF. Given the recent and still poorly understood nature of the disease, further studies with larger samples and longer follow-up are necessary to deepen the understanding of its evolution, cardiovascular impact, and potential future complications.

## Funding sources

This research did not receive any specific grant from funding agencies in the public, commercial, or not-for-profit sectors.

## Data availability statement

The data that support the findings of this study are available from the corresponding author.

## Conflicts of interest

The authors declare no conflicts of interest.

## Appendix Supplementary materials

Image, application 1

## Footnotes

Supplementary material associated with this article can be found in the online version at doi:10.1016/j.jped.2025.101461.
