# Supplementary material for: Cardiac manifestations in children and adolescents diagnosed with pediatric multisystem inflammatory syndrome related to COVID-19
Source: J Pediatr (Rio J). 2025 Oct 31;101(6):101461. doi: 10.1016/j.jped.2025.101461 (PMC12616072; doi:10.1016/j.jped.2025.101461)

**JPED-D-25-00308_Supplementary Material**

**Table S1** Clinical characteristics of the study population at admission.

| Signs and symptoms at admission: n, (%) |  |  | Total patients (n = 36) |
| --- | --- | --- | --- |
| Tachycardia |  |  | 8 (22.2) |
| Prostration |  |  | 8 (22.2) |
| Hypotension |  |  | 5 (13.9) |
| Hypoperfusion/shock |  |  | 4 (11.1) |
| Irritability |  |  | 3 (8.3) |
| Hypertension |  |  | 2 (5.6) |
| SO_2_ < 95% |  |  | 2 (5.6) |
| Weight loss |  |  | 2 (5.6) |
| Clinical manifestations: n, (%) |  |  |  |
| Fever |  |  | 36 (100) |
| Mucocutaneous |  |  | 32 (88.9) |
| Respiratory |  |  | 27 (75) |
| Gastrointestinal |  |  | 26 (72.2) |
| Lymphatic |  |  | 21 (58.3) |
| Cardiovascular |  |  | 13 (36.1) |
| Musculoskeletal |  |  | 9 (25) |
| Neurological |  |  | 9 (25) |
| Genitourinary |  |  | 6 (16.7) |

Values are presented as n (%) for categorical variables.

n, number of individuals; SO_2_, Oxygen saturation.

**Table S2** Laboratory findings at admission.

| Laboratory findings: n, (%) |  |  | Total patients (n = 36) |
| --- | --- | --- | --- |
| D-Dimer > 501 ng/m |  |  | 28/31 (90.3) |
| LDH > 246 U/L |  |  | 23/28 (82.1) |
| CRP > 10 mg/L |  |  | 28 (77.8) |
| ESR > 40 mm |  |  | 25/33 (75.8) |
| Ferritin > 150 ng/mL |  |  | 13/20 (65) |
| Fibrinogen > 400 mg/dL |  |  | 17/28 (60.7) |
| Leukocytosis > 15,000 /μL |  |  | 16 (44.4) |
| AST > 36 M / > 59 F U/L |  |  | 14/35 (40) |
| Anemia < 10 g/dl |  |  | 12 (33.3) |
| Thrombocytosis > 450,000 /μL |  |  | 10 (27.8) |
| Sodium < 135 mmol/L |  |  | 7/33 (21.2) |
| ALT > 51 U/L |  |  | 7/35 (20) |
| Thrombocytopenia < 150,000 /μL |  |  | 6 (16.7) |
| Albumin < 3 g/dL |  |  | 5/33 (15.2) |
| Fibrinogen < 200 mg/dL |  |  | 4/28 (14.3) |
| Increased creatinine |  |  | 4/32 (12.5) |
| aPTT > 1.3 |  |  | 2/29 (6.9) |
| INR > 1.3 |  |  | 2/32 (6.3) |
| Lymphopenia < 1,500 /μL |  |  | 2/35 (5.7) |
| Urea > 50 mg/dL |  |  | 1/31 (3.2) |
| Leukopenia < 4,000 /μL |  |  | 1 (2.8) |

Values are presented as n (%) for categorical variables.

n, number of individuals; M, Male; F, Female; LDH, Lactate Dehydrogenase; CRP, C-Reactive Protein; ESR, Erythrocyte Sedimentation Rate; AST, Aspartate Aminotransferase; ALT, Alanine Aminotransferase; aPTT, activated Partial Thromboplastin Time; INR, International Normalized Ratio.

**Table S3** Laboratory test results at admission.

| Variation of laboratory parameters at admission | Mean ± SD, or median (IQR) |
| --- | --- |
| Hemoglobin (g/dL)  mean (±SD) | 10.5 ± 1.5 |
| Leukocytes (/μL)  median (IQR) | 12,495 (8,730) |
| Lymphocytes (/μL)  median (IQR) | 3,444 (2,707) |
| Platelets (× 10³/μL)  mean (±SD) | 364,389 ± 206,418.3 |
| CRP (mg/L)  median (IQR) | 52.2 (152.33) |
| ESR (mm)  mean (±SD) | 66 ± 37.6 |
| D-Dimer (ng/mL)  median (IQR) | 1,869 (2,659) |
| INR  median (IQR) | 1.14 (0.22) |
| aPTT  median (IQR) | 1.3 (0.16) |
| Fibrinogen (mg/dL)  mean (±SD) | 482.25 ± 228.5 |
| Ferritin (ng/mL)  median (IQR) | 173 (203.6) |
| LDH (U/L)  median (IQR) | 281 (125) |
| Urea (mg/dL)  median (IQR) | 16 (17) |
| Creatinine (mg/dL)  median (IQR) | 0.25 (0.27) |
| Albumin (g/dL)  mean (±SD) | 3.5 ± 0.57 |
| AST (U/L)  median (IQR) | 34 (23) |
| ALT (U/L)  median (IQR) | 23 (30) |
| Sodium (mmol/L)  mean (±SD) | 137.4 ± 3.3 |

To assess the distribution of continuous variables, the Shapiro-Wilk normality test was applied. Median with an interquartile range was used for variables with non-normal distribution and mean ± SD for variables with normal distribution.

SD, Standard Deviation; IQR, Interquartile range; LDH, Lactate Dehydrogenase; CRP, C-Reactive Protein; ESR, Erythrocyte Sedimentation Rate; AST, Aspartate Aminotransferase; ALT, Alanine Aminotransferase; aTTP, Activated Partial Thromboplastin Time; INR, International Normalized Ratio.

**Table S4** Clinical and electrocardiographic cardiovascular findings at admission.

| Clinical manifestations: n, (%) |  |  | Total patients  (n = 36) |
| --- | --- | --- | --- |
| Arrhythmias |  |  | 9 (25) |
| Hypotension/Shock |  |  | 5 (13.9) |
| Pericarditis/pericardial effusion |  |  | 3 (8.3) |
| Hypertension |  |  | 2 (5.6) |
| Heart failure |  |  | 2 (5.5) |
| Thrombosis |  |  | 1 (2.8) |
| Valvulitis |  |  | 0 (0) |
| Electrocardiographic findings: n, (%) |  |  |  |
| Altered ECG |  |  | 9 (25) |
| Tachyarrhythmias |  |  | 8 (22.2) |
| First-degree atrioventricular block |  |  | 1 (2.8) |
| Left Atrial Enlargement |  |  | 1 (2.8) |

Values are presented as n (%) for categorical variables.

n, number of individuals; ECG, Electrocardiogram.

**Table S5** TAPSE, MAPSE, and E/E’ ratio values at one-year follow-up visit.

| Echocardiographic parameters |  |  | One-year follow-up |
| --- | --- | --- | --- |
| TAPSE  Z score: median (IQR) |  |  | 1.45 (2.46) |
| Reduced: n, (%) |  |  | 0/21 (0) |
| Increased: n, (%) |  |  | 7/21 (33.3) |
| MAPSE  Z score: mean (±SD) |  |  | 2.45 ± 2.4 |
| Reduced: n, (%) |  |  | 0/21 (0) |
| Increased: n, (%) |  |  | 11/21 (52) |
| E/E’ ratio  Z score: mean (±SD) |  |  | -1.42 ± 0.89 |
| Reduced: n, (%) |  |  | 6/20 (30) |
| Increased: n, (%) |  |  | 0/20 (0) |

Values are presented as n (%) for categorical variables.

To assess the distribution of continuous variables, the Shapiro-Wilk normality test was applied. Median with an interquartile range was used for variables with non-normal distribution and mean ± SD for variables with normal distribution.

n, number of individuals; SD, Standard Deviation; IQR, Interquartile range; TAPSE, Tricuspid Annular Plane Systolic Excursion; MAPSE, Mitral Annular Plane Systolic Excursion.

**Table S6** Treatment administered to patients diagnosed with MIS-C.

| Treatment administered |  |  | Total patients (n = 36) |
| --- | --- | --- | --- |
| ASA: n, (%) |  |  | 36 (100) |
| Time to aspirin initiation  days: median (IQR) |  |  | 10.5 (10) |
| Intravenous human immunoglobulin: n, (%) |  |  | 29 (80.6) |
| Time to immunoglobulin initiation  days: median (IQR) |  |  | 8 (7) |
| Oral corticosteroids: n, (%) |  |  | 25 (69.4) |
| Time to oral corticosteroid initiation  days: median (IQR) |  |  | 11.5 (7) |
| Intravenous corticosteroids: n, (%) |  |  | 15 (41.7) |
| Time to initiation of intravenous corticosteroids  days: median (IQR) |  |  | 9 (8) |
| Fluid resuscitation or need for IV hydration: n, (%) |  |  | 11 (30.6) |
| Diuretic: n, (%) |  |  | 5 (13.9) |
| Inotropic support: n, (%) |  |  | 3 (8.3) |
| Pulse therapy: n, (%) |  |  | 3 (8.3) |
| Mechanical ventilation: n, (%) |  |  | 2 (5.6) |
| Antihypertensive: n, (%) |  |  | 1 (2.8) |

Values are presented as n (%) for categorical variables.

To assess the distribution of continuous variables, the Shapiro-Wilk normality test was applied. Median with an interquartile range was used for variables with non-normal distribution and mean ± SD for variables with normal distribution.

n, number of individuals; IQR, Interquartile range; SD, Standard Deviation; ASA, Acetylsalicylic acid; IV, Intravenous.

**Table S7** Comparative table of clinical data and treatment of patients diagnosed with MIS-C with and without cardiovascular involvement.

| Variables | No cardiovascular involvement  (n = 21) | With cardiovascular involvement  (n = 15) | p-value |
| --- | --- | --- | --- |
| Male: n, (%) | 14 (66.6) | 11 (73.3) | 0.73^1^ |
| Age at diagnosis:  years: median (IQR) | 2,9 (3.1) | 1,6 (3.4) | 0.6^3^ |
| Comorbidities: n, (%) | 2 (9.5) | 5 (33.3) | 0.1^1^ |
| ICU admission: n, (%) | 0 (0) | 4 (26.6) | **0.02^1^** |
| Ventilatory support: n, (%) | 0(0) | 2 (13.3) | 0.17^1^ |
| Inotropic support: n, (%) | 0 (0) | 3 (20) | 0.06^1^ |
| Volume support: n, (%) | 3 (14.3) | 8 (53.3) | **0.02^1^** |
| Mucocutaneous alterations: n, (%) | 18 (85.7) | 14 (93.3) | 0.6^1^ |
| Musculoskeletal alterations: n, (%) | 4 (19) | 5 (33.3) | 0.4^1^ |
| Gastrointestinal alterations: n, (%) | 14 (66.6) | 12 (80) | 0.4^1^ |
| Lymphatic involvement: n, (%) | 10 (47.6) | 11 (73.3) | 0.12^2^ |
| Respiratory alterations: n, (%) | 15 (71.4) | 12 (80) | 0.7^1^ |
| Neurological alterations: n, (%) | 4 (19) | 5 (33.3) | 0.4^1^ |
| Genitourinary alterations: n, (%) | 2 (9.5) | 4 (26.6) | 0.2^1^ |
| Use of intravenous immunoglobulin: n, (%) | 18 (85.7) | 11 (73.3) | 0.4^1^ |
| Use of oral corticosteroids: n, (%) | 15 (71.4) | 10 (66.6) | 1^1^ |
| Use of intravenous corticosteroids: n, (%) | 6 (28.6) | 9 (60) | 0.06^2^ |
| Pulse therapy: n, (%) | 0 (0) | 3 (20) | 0.06^1^ |
| Use of diuretics: n, (%) | 0 (0) | 5 (33.3) | **0.008^1^** |

Values are presented as n (%) for categorical variables.

To assess the distribution of continuous variables, the Shapiro-Wilk normality test was applied. Median with an interquartile range was used for variables with non-normal distribution and mean ± SD for variables with normal distribution.

n, number of individuals; IQR, Interquartile range; SD, Standard Deviation; ICU, Intensive Care Unit.

^1^ Fisher's exact test.

^2^ Chi-squared test.

^3^ Wilcoxon rank-sum test.

**Table S8** Comparative table of laboratory data of patients diagnosed with MIS-C with and without cardiovascular involvement.

| Variables: n, (%) | No cardiovascular involvement  (n = 21) | With cardiovascular c involvement  (n = 15) | p-value |
| --- | --- | --- | --- |
| Anemia < 10 g/dL | 6 (28.6) | 6 (40) | 0.5^2^ |
| Leukocytosis > 15,000 /μL | 9 (42.8) | 7 (46.6) | 0.8^2^ |
| Leukopenia < 4,000 /μL | 0 (0) | 1 (6.6) | 0.4^1^ |
| Lymphopenia < 1,500 /μL | 0 (0) | 2 (13.3) | 0.1^1^ |
| Thrombocytosis > 450,000 /μL | 5 (23.8) | 5 (33.3) | 0.7^1^ |
| Thrombocytopenia < 150,000 /μL | 2 (9.5) | 4 (26.6) | 0.2^1^ |
| CRP > 10 mg/L | 14 (66.6) | 14 (93.3) | 0.1^1^ |
| ESR > 40 mm | 16 (76.2) | 9 (60) | 0.2^1^ |
| D-Dimer > 501 ng/m | 15 (71.4) | 13 (86.6) | 1^1^ |
| Fibrinogen > 400 mg/dL | 12 (57.1) | 5 (33.3) | 0.1^1^ |
| Ferritin > 150 ng/mL | 6 (28.6) | 7 (46.6) | 0.1^1^ |
| AST > 36 M / > 59 F U/L | 5 (23.8) | 9 (60) | **0.036^2^** |
| ALT > 51 U/L | 2 (9.5) | 5 (33.3) | 0.1^1^ |
| Urea > 50 mg/dL | 0 (0) | 1 (6.6) | 0.4^1^ |
| Increased creatinine | 3 (14.3) | 1 (6.6) | 0.6^1^ |
| Sodium < 135 mmol/L | 3 (14.3) | 4 (26.6) | 0.4^1^ |
| INR > 1.3 | 0 (0) | 2 (13.3) | 0.1^1^ |
| aPTT > 1.3 | 1 (4.8) | 1 (6.6) | 1^1^ |
| Albumin < 3 g/dL | 1 (4.8) | 4 (26.6) | 0.1^1^ |
| LDH > 246 U/L | 11 (52.4) | 12 (80) | 0.3^1^ |

No MIS-C patient had serum sodium greater than 155 mmol/L

Values are presented as n (%) for categorical variables.

n, number of individuals; LDH, Lactate Dehydrogenase; CRP, C-Reactive Protein; ESR, Erythrocyte Sedimentation Rate; AST, Aspartate Transaminase; ALT, Alanine Transaminase; aPTT, Activated Partial Thromboplastin Time; INR, International Normalized Ratio.

^1^ Fisher's exact test.

^2^ Chi-squared test.

^3^ Wilcoxon rank-sum test.

**Figure S1** Bland-Altman plot showing intra- (A) and inter-observer (B) variation in the measurement of coronary artery diameter. M – Mean; SD - Standard Deviation.


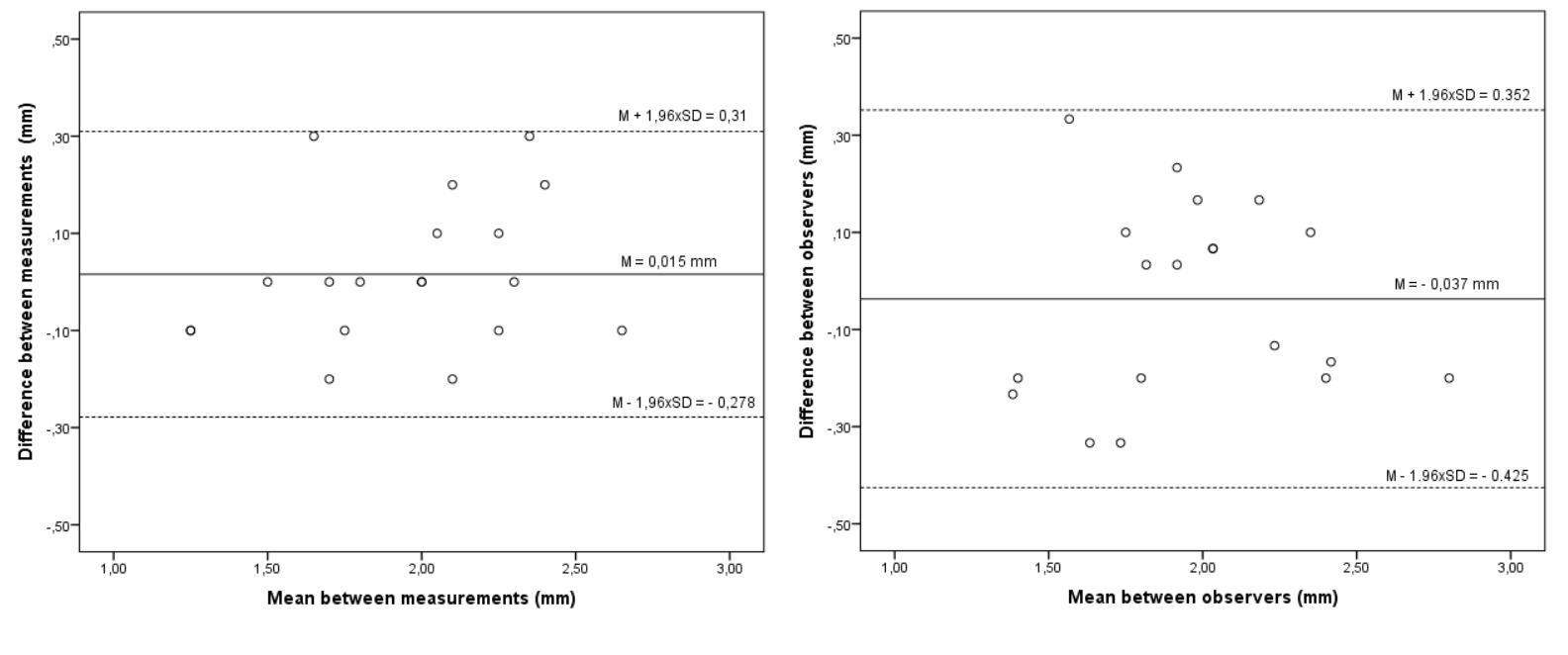

Supplement: Supplementary file 1 [file mmc1.docx]
